# Accuracy of clinical neurological examination in diagnosing lumbo-sacral radiculopathy: a systematic literature review

**DOI:** 10.1186/s12891-016-1383-2

**Published:** 2017-02-23

**Authors:** Nassib Tawa, Anthea Rhoda, Ina Diener

**Affiliations:** 1 Department of Rehabilitative Sciences, College of Health Sciences, Jomo Kenyatta University of Agriculture & Technology, P. O. Box 62 000 00200, Nairobi, Kenya; 2 Division of Physiotherapy, Faculty of Medicine and Health Sciences, Stellenbosch University, Private Bag X1 7602, Matieland, South Africa; 3 Department of Physiotherapy, Faculty of Community and Health Sciences, University of the Western Cape, Private Bag X 17, 7535 Bellville, Republic of South Africa

**Keywords:** Lumbar radiculopathy, Diagnostic accuracy, Clinical neurological examination

## Abstract

**Background:**

Lumbar radiculopathy remains a clinical challenge among primary care clinicians in both assessment and diagnosis. This often leads to misdiagnosis and inappropriate treatment of patients resulting in poor health outcomes, exacerbating this already debilitating condition. This review evaluated 12 primary diagnostic accuracy studies that specifically assessed the performance of various individual and grouped clinical neurological tests in detecting nerve root impingement, as established in the current literature.

**Methods:**

Eight electronic data bases were searched for relevant articles from inception until July 2016. All primary diagnostic studies which investigated the accuracy of clinical neurological test (s) in diagnosing lumbar radiculopathy among patients with low back and referred leg symptoms were screened for inclusion. Qualifying studies were retrieved and independently assessed for methodological quality using the ‘Quality Assessment of Diagnostic tests Accuracy Studies’ criteria.

**Results:**

A total of 12 studies which investigated standard components of clinical neurological examination of (sensory, motor, tendon reflex and neuro-dynamics) of the lumbo-sacral spine were included. The mean inter-observer agreement on quality assessment by two independent reviewers was fair (k = 0.3 – 0.7).

The diagnostic performance of sensory testing using MR imaging as a reference standard demonstrated a sensitivity (confidence interval 95%) 0.61 (0.47-0.73) and a specificity of 0.63 (0.38-0.84). Motor tests sensitivity was poor to moderate, ranging from 0.13 (0.04-0.31) to 0.61 (0.36-0.83). Generally, the diagnostic performance of reflex testing was notably good with specificity ranging from (confidence interval 95%) 0.60 (0.51-0.69) to 0.93 (0.87-0.97) and sensitivity ranging from 0.14 (0.09-0.21) to 0.67 (0.21-0.94). Femoral nerve stretch test had a high sensitivity of (confidence interval 95%) 1.00 (0.40-1.00) and specificity of 0.83 (0.52-0.98) while SLR test recorded a mean sensitivity of 0.84 (0.72-0.92) and specificity of 0.78 (0.67-0.87).

**Conclusions:**

There is a scarcity of studies on the diagnostic accuracy of clinical neurological examination testing. Furthermore there seem to be a disconnect among researchers regarding the diagnostic utility of lower limb neuro-dynamic tests which include the Straight Leg Raise and Femoral Nerve tests for sciatic and femoral nerve respectively. Whether these tests are able to detect the presence of disc herniation and subsequent nerve root compression or hyper-sensitivity of the sacral and femoral plexus due to mechanical irritation still remains debatable.

## Background

Lumbo-sacral radiculopathy, a substantial cause of disability and morbidity, represents one distinct presentation of low back-related leg pain, which constitutes between 23% - 57% of LBP cases [1]. Lumbo-sacral radiculopathy refers to a pathologic process involving the lumbo-sacral nerve roots causing radicular symptoms into a lower extremity [2], which may or may not be accompanied by other radicular irritation symptoms and/or symptoms of decreased function [3]. Lumbar IVD protrusion is the most common cause underlying nerve root irritation and subsequent radiculopathy [1–3]. However, other mechanical factors including, lumbar vertebrae osteophytes, lumbar facet joint hypertrophy or ligamentum flavum hypertrophy may also cause lumbar nerve root compression [3]. Radicular symptoms may also be primarily caused by inflammatory reactions of the neural or surrounding musculo-articular structures [4], hence suggesting that lumbar radiculopathy is not always mechanicall*y* mediated, and that mechanical nerve root compression on its own does not necessarily determine radicular symptoms as seen on positive MRI findings on asymptomatic subjects [5].

In clinical practice, the diagnosis of lumbo-sacral radiculopathy involves the use of various tools and procedures including neuropathic pain screening, clinical neurological examination, electro-diagnosis, nerve root blockage and radiological imaging [3–5]. Clinical neurological tests include sensory, motor, reflex, neuro-dynamic and nerve trunk palpation procedures designed to assess the physiological and bio-mechanical status of specific lumbar nerve roots thought to be responsible for the patient's signs and symptoms [5]. Determination of the presence or absence of radiculopathy is dependent upon the examiner's awareness of clinical signs and symptoms, physical examination, knowledge of possible pathology, mechanisms of injury and ability to perform the tests correctly [6–8]. The clinical usefulness of neurological examination tests is largely determined by the accuracy with which they determine the presence or abscence of the suspected patho-neuro-physiology.

MRI is frequently utilized in detecting nerve root compression, one of the many causes of radiculopathy [4, 9]. While the accuracy of MRI in detecting alterations in both the anatomy and tissue properties is well established, the relationship between the detected anatomical abnormalities and clinical history and patients outcomes remain controversial [6].

Although MRI is being used as a diagnostic tool of choice by clinicians in practice and a gold standard by researchers in primary diagnostic accuracy studies [10, 11], there are several limitations proposed in the literature. One, MRI embraces the patho-anatomical model yet radiculopathy is not always mechanicall*y* mediated by IVD nerve root compression as earlier reported [12]. Two, there is not an acceptable gold standard diagnostic tool to which MRI can be compared [13–15] This is because, even though conventional electro-diagnostic procedures are sometimes used as gold standard for detecting nerve involvement, experts argue that they leave the function of small caliber afferent fibers unexplored, and therefore, there is no basis for positive findings [7–9]. Current perception threshold testing [7], electro-myelography [8], and nerve root blocks [9] on the other hand are used mainly to confirm symptomatic structures.

Early and accurate diagnosis of lumbar radiculopathy is crucial to ensure target-specific treatment and avoid chronicity, disability and work loss [14, 15] and clinical neurological examination forms a vital component of the initial diagnostic work-up for patients with clinical suspicion of lumbar radiculopathy. Clinical neurological examination tests could be used to discriminate patients with radiculopathy distinct from other low back pain sub-types like non-specific low back pain of somatic origin, lumbar facet or intervetebral joint derangement disorder. These tests are easy to perform, cost-effective and run a relatively very low health risk to patients. It is therefore imperative to identify those which have a reported acceptable diagnostic sensitivity and/or specificity through a structured systematic review. The available systematic literature reviews which have been published recently have an evident variation in case definition of lumbar radiculopathy and have also focused on detection of disc herniation or protrusion as the only cause of nerve root compression and subsequent radiculopathy [10, 11]. Different from this trend and for the purposes of this review, our operational clinical definition for lumbo-sacral radiculopathy was: “Objective loss of sensory and motor function with or without accompanied spinal and/or referred leg pain following a mechanical or bio-chemical dysfunction of lumbar and sacral spinal nerve roots and their associated dorsal root ganglions (DRGs)”. This review therefore aimed at determining the accuracy of clinical neurological tests in diagnosing lumbo-sacral radiculopathy.

## Methods

This review was conducted using the diagnostic tests accuracy (DTA) protocol [10].

### Search strategy

A comprehensive search was conducted up until July 2016 to identify relevant studies in various electronic databases including MEDLINE, CINAHL, Biomed Central, Science Direct, Springerlink, Google scholar, Pubmed, and Embase. No publication date limitation was imposed thus all databases were searched since inception. The search was performed by one reviewer (NT) who also conducted complementary hand searching of field- and topic-relevant journals including reference lists of potentially relevant articles.

Study selection was independently performed by two reviewers (NT and ID) using the Patient, Intervention, Comparison, Outcome (PICO) analysis [16] and disagreements were resolved through discussion and the opinion of a third reviewer (AR). A study was selected if; it used patients with clinical signs and symptoms suggestive of lumbar radiculopathy, assessed the accuracy of any aspect of clinical neurological examination as an index diagnostic test and used magnetic resonance imaging, CT myelography, electro-diagnostics, spinal nerve root block or intra-operative findings as a reference. Based on the information in the title and abstract, 12 studies were prequalified as potentially relevant and were retrieved as full articles for further review.

Two reviewers (NT and AR) independently assessed the quality of all included studies using the Quality Assessment of Diagnostic Accuracy Studies (QUADAS) criteria [17]. Scoring disagreements between the two reviewers were resolved by a discussion arbitrated by the third reviewer (ID) until a consensus was reached. QUADAS is a 12-item methodological checklist which mainly focuses on the subjects’ description, index test, comparator test and the examiners (Table 1). Each of the included studies was separately assessed for each of the 12 items. Studies were scored as ‘positive’ (+), when the described methodology was of acceptable quality, as ‘negative’ (−), when the described methodology was not of acceptable quality, and ‘not sure’ (?), when the methodology was inadequately described. A cumulative percentage across all included studies was then scored per item, and per study.

**Table 1:** QUADAS scores of included studies

Author (year)	Criteria number												
	1	2	3	4	5	6	7	8	9	10	11	12	Total (%)
Iversen et al. (2013)	+	+	+	?	-	+	+	+	+	+	-	+	75
Suri et al. (2011) [21]	+	+	+	?	+	+	+	?	+	+	_	+	75
Trainor & Pinnington (2011) [16]	+	+	?	_	+	+	_	?	+	_	_	+	50
Coster et al. (2010) [7]	+	?	+	?	+	+	+	?	_	+	_	_	50
Suri et al. (2010) [15]	+	+	+	?	+	+	+	?	+	+	_	+	75
Bertilson et al. (2010) [14]	+	+	+	+	+	+	+	+	+	+	_	+	92
Lee-Robinson and Lee (2010) [2]	+	+	?	?	+	+	+	+	+	?	_	+	67
Majlesi (2008) [20]	+	?	+	?	+	+	+	?	_	+	_	**_**	50
Rabin (2007) [19]	+	+	?	**_**	+	+	+	+	+	+	_	+	75
Vroomen et al. (2002) [10]	+	+	?	**_**	+	+	+	+	+	+	_	+	75
Haldeman (1998) [18]	+	+	?	**_**	+	+	+	+	+	+	_	+	75
Albeck (1996) [13]	+	?	+	?	+	+	+	?	_	+	_	+	58
% of maximum	100	72	55	9	100	100	90	45	72	82	0	82	

### Data extraction

The first reviewer (NT) independently extracted data from the original included studies using a standardized self-developed form which covered: Participants (number, age, gender, clinical characteristics, clinical setting), examiners (profession and expertise) and clinical test (s). Data from each included study was retrieved to allow calculation of sensitivity and specificity values of the target index tests. The reviewers extracted, or where unavailable re-calculated the common parameters of diagnostic test accuracy including; sensitivity, specificity, positive likelihood ratio (+LR), negative likelihood ratio (−LR) and diagnostic odds ratios (DOR). Also, true positive, false positive, true negative and false negatives of each investigated index test is presented. A meta-analysis was not conducted given the heterogenity of included studies in this review.

## Results

**Fig. 1:**
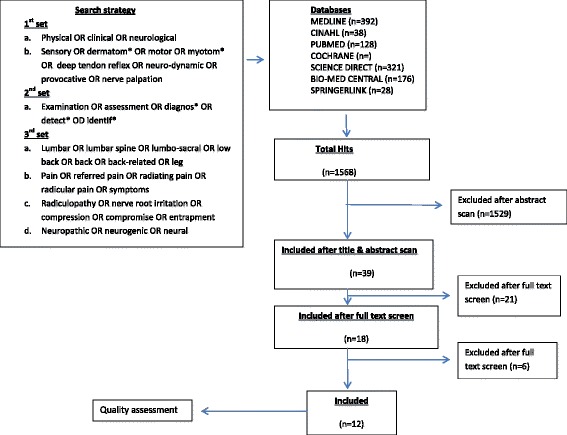
Search history

The search on relevant electronic data bases retrieved a total of 1568 articles (Fig. 1) by the first hit of the key terms and the mesh terms. After screening the title, key words and abstract of all articles and removal of duplicates, 39 articles were selected as potentially suitable for inclusion and were retrieved as full articles for further analysis. Out of the 39, 24 were selected from those that were generated by the entry of the key terms while 15 were selected from the output of the mesh terms. Full screening of the 39 articles was independently done by two reviewers (NT & ID) using a PICO analysis and disagreements were resolved through adjudication by a third reviewer (AR). Twenty-eight studies were further excluded for not meeting the inclusion criteria. An additional reference hand-searching of all included studies and subject specific journals was done by one reviewer (NT) but did not yield any more relevant studies. A total of 12 studies whose characteristics are summarized in Table 2 (Albeck 1996 [13], Haldeman et al. 1998 [18], Vroomen et al. 2002 [10], Rabin 2007 [19], Majlesi et al. 2008 [20], Bertilson et al. 2010 [14], Lee-Robinson et al. 2010 [2], Coster et al. 2010 [7], Suri et al. 2010 [15], Suri et al. 2011 [21], Trainor and Pinnington 2011 [16], Iversen et al. 2013) met the inclusion criteria (Published as a full article in a peer-reviewed journal, in English; Evaluated the sensitivity and/or specificity of clinical neurological test (s) in diagnosing lumbar/sacral radiculopathy; Incorporated a comparator test (s); Study subjects presented with clinical signs and symptoms consistent with lumbo-sacral radiculopathy as diagnosed by the referring clinicians). Of the 12 studies included in this review, 11 were cohorts while 1 was a case control study.

**Table 2:** Characteristics of included studies

Author (year) Country	Sample size (gender, age)	Setting (period of recruitment)	Patients’ description	Examiners	Index tests
Iversen et al. (2013) Norway	*N* = 116 Male = 68 Female = 48 Mean age = 42	Out-patient multi-disciplinary back clinics	History & clinical presentation suggestive of chronic lumbar radiculopathy	Orthopaedic manual physiotherapists and neurologists	FNS, SLR, sensory, motor, knee reflex, ankle reflex
Suri et al. (2011) [21] USA	*N* = 54 Male =28 Female = 26 Mean age = 54	Hospital spine centre (January 2008 – March 2009)	Lower extremity radiating pain of	Physiatrists specialized in spine care	SLR, Crossed SLR, FNST, sensory, motor, patella & Achilles reflexes
Trainor & Pinnington (2011) [16] UK	*N* = 16 Male =7 Female = 9 Mean age = 49	Orthopedic spinal clinic (6 months)	Pain radiating into one or both legs distal to the groin or gluteal fold Distribution of pain in dermatomal pattern	Physiotherapists	Slump knee bend test
Coster et al. (2010) [7] Netherlands	*N* = 202 Male =92 Female = 110 Mean age = 46	Neurology department (January 2006 – March 2007)	Subjects referred by general practitioners with clinical suspicion of Lumbo-Sacral Radicular Syndrome (LSRS)	Neuro-physiologist	Sensory, motor, reflex and SLR test
Suri et al. (2010) [15] USA	*N* = 51 (independent group) Male =40 Female = 11 Mean age = 54	Hospital spine centre (January 2008 – March 2009)	Patients presenting with lower extremity radiating pain and MRI-visible lumbar disk herniation.	Physiatrists specialized in spine care	Sensory (soft touch & pin prick), motor (heel raise & sit-to-stand), reflex and neural provocation
Bertilson et al. (2010) [14] Sweden	*N* = 61 Male =12 Female = 49 Mean age = 60	Radiology clinic (February - September 2004)	Patients referred for lumbar spine MRI	Orthopaedic surgeon & certified radiologist	Sensory (soft touch & pain prick), motor (hypotrophy), tendon reflex, tender point palpation
Lee-Robinson (2010) [2] USA	*N* = 70 Male =31 Female = 39 Mean age = 65	Electro-diagnosis, physical medicine & rehabilitation clinic (January to October 2009)	Patients with low back pain and radicular lower extremity symptoms of weakness, numbness, and pain and abnormal lumbar MRI findings	Physician (specialist in electro-diagnostics & physical medicine & rehab)	Ankle reflex, pin wheel examination, motor testing
Majlesi (2008) [20] Turkey	*N* = 180 Male = Female = Mean age =	Neuro-surgery department (January – June 2005)	Patients with complaints suggestive of lumbar disc herniation with low back, leg, or low back and leg pain	Neuro-surgeons	Slump and SLR tests
Rabin (2007) [19] USA	*N* = 38 (MRI +) Male =30 Female = 8 Mean age = 38	Neuro- and orthopedic surgery clinic	Low back pain or paraesthesia radiationg below the knee	Unclear	Seated SLRT and supine SLRT
Vroomen et al. (2002) [10] Netherlands	*N* = 58 (MRI +) Male = ? Female = ? Mean age = ?	Neurology department	Patients referred to the neurology department with a new episode of pain radiating into the leg below the gluteal fold.	Neuro-radiologist	Paresis, finger floor distance, tendon reflexes, sensory,
Haldeman (1998) [18] USA	*N* = 100 Male = ? Female = ? Mean age = ?	Neurology and surgery	Patients with complaints of low-back pain and leg pain, consistent with a diagnosis of sciatica	Certified neuro-radiologist and orthopaedic surgeon	SLRT, motor, sensory, reflex
Albeck (1996) [13] Denmark	*N* = 80 Male =48 Female = 32 Mean age = 40	Neuro-surgery clinic	Mono-radicular pain from L5 or S1	Neuro-surgeon	sensory, motor, tendon reflexes

The clinical neurological examination tests assessed by the included studies were the standard sensory (soft touch and pin prick), motor (functional tests and resisted isometric contractions), deep tendon reflex (patella) and neuro-dynamic (Straight Leg Raise and Femoral nerve) tests. MR imaging was used as a reference standard in 8 of the included studies while 2 studies used EMG, one electro-diagnostics and CT, and the other one intra-operative findings. Eleven studies were carried out in secondary and tertiary care settings while one was a primary care diagnostic study. The search history is displayed in Fig. 1.

The QUADAS scores for each of the 12 included studies across all QUADAS items ranged from 50% – 92% (vertically) and the scores for all included studies per QUADAS item ranged from 0 – 100% (horizontally). The Bertilson et al. (2010) [14] study had the highest score of 92% across all QUADAS items followed by Suri et al. 2011 [21], Rabin et al. 2007 [19], Vroomen et al. 2002 [10], Haldeman et al. 1998 [18] and Iversen et al. 2013 while the Albeck 1996 [13] study had the lowest score of 58%. All studies fulfilled items 1, 5 and 6 which concern a representative spectrum of study subjects, verification bias and clear explanation of index test execution respectively; while none of the 12 studies met criteria 11 on reporting uninterpretable or intermediate index test results. The scores are displayed in Table 1.

### Summary of diagnostic accuracy of individual tests

#### Sensory tests

Accuracy of sensory tests in identifying nerve root impingement was evaluated in 5 studies and is summarized in Table 3. The various aspects, whose diagnostic performance was assessed, included hypo-aesthesia, paraesthesia and anaesthesia. The actual procedure was not well reported in most of the studies. Dermatome maps were used to guide the procedure. The Albeck (1996) [13] study which was the oldest among the 5 reported the best sensitivity (confidence interval 95%) 0.61 (0.47-0.73) with a relatively moderate specificity of (confidence interval 95%) 0.63 (0.38-0.84). This seemingly high sensitivity of sensory test in the Albeck (1996) [13] study compared to the other 4 studies which evaluated sensibility to touch using MR imaging as a reference standard may be attributed to the fact that patients who are scheduled for surgery are routinely carefully selected compared to those whom surgey is not contemplated. Hence the probability of a positive index test results becomes relatively higher in the surgical than imaging group. A rather recent study by Suri et al. (2010) [15] presented the best specificity for sensibility testing in detecting nerve root impingement at (confidence interval 95%) 0.96 (0.82-1.00).

**Table 3:** Diagnostic accuracy of sensory tests

Author (year)	Reference standard	Sensitivity (95% CI)	Specificity (95% CI)	+ LR	- LR
Suri et al. (2010) [15] L2	MRI	0.08 (0.01-0.27)	0.96 (0.82-1.00)	2.0	1.0
L3	MRI	0.17 (0.05-0.37)	0.96 (0.82-1.00)	4.3	1.2
L4	MRI	0.17 (0.05-0.37)	1.00 (0.88-1.00)	0.2	1.2
L5	MRI	0.13 (0.03-0.34)	0.82 (0.63-0.94)	0.7	0.9
S1	MRI	0.08 (0.01-0.27)	0.79 (0.59-0.92)	0.4	0.9
Iversen et al. (2013)	MRI & CT	0.33 (0.06-0.79)	0.88 (0.81-0.93)	2.8	1.3
Bertilson et al. (2010) [14] (L4)	MRI	0.07 (0.01-0.22)	0.81 (0.63-0.93)	0.4	0.9
L5		0.17 (0.06-0.35)	0.58 (0.39-0.75)	0.4	0.7
S1		0.20 (0.08-0.39)	0.84 (0.66-0.95)	1.3	1.1
Albeck (1996) [13]	Surgery	0.61 (0.47-0.73)	0.63 (0.38-0.84)	1.6	1.6
Vroomen et al. (2002) [10]	MRI	0.14 (0.09-0.21)	0.93 (0.87-0.97)	2.0	1.1

### Motor tests

Six of the included 12 studies evaluated the diagnostic accuracy of motor tests using functional tests and resisted isometric contraction to determine paresis or muscle weakness. None of the studies reported elaborate information regarding execution and criteria for positivity. Generally, motor tests across all primary diagnostic studies reported a relatively poor sensitivity. The highest (confidence interval 95%) 0.61 (0.36-0.83) was for great toe extension test in detecting L5 nerve root impingement reported in the Suri et al. (2011) [21] study.

Similarly, dorsiflexion and great toe extension had the highest specificity (confidence interval 95%) 0.93 (0.87-0.97), as reported in the only primary care study Vroomen et al. (2002) [10], however, this was not specific to any segmental nerve root level. The diagnostic parameters of motor tests are summarized in Table 4.

**Table 4:** Diagnostic accuracy of motor tests

Author (year)	Reference standard	Sensitivity (95% CI)	Specificity (95% CI)	+LR	-LR
Suri et al. (2010) [15]	MRI	0.39 (0.32-0.52)	0.83 (0.78-0.87)	2.3	1.4
Iversen et al. (2013)	MRI & CT	O.33 (0.06-0.97)	0.68 (0.59-0.76)	1.0	1.0
Suri et al. (2011) [21] L3	MRI	0.50 (0.19-0.81)	0.77 (0.62-0.89)	2.2	1.5
L4	MRI	0.54 (0.25-0.81)	0.80 (0.65-0.91)	2.7	1.7
L5	MRI	0.61 (0.36-0.83)	0.86 (0.71-0.95)	4.4	2.2
S1	MRI	0.29 (0.10-0.56)	0.97 (0.85-1.00)	1.0	1.4
Albeck (1996) [13]	Surgery	0.34 (0.23-0.48)	0.47 (0.24-0.71)	0.6	0.7
Vroomen et al. (2002) [10]	MRI	0.27 (0.20-0.35)	0.93 (0.87-0.97)	3.9	1.3
Bertilson et al. (2010) [14] L4	MRI	0.13 (0.04-0.31)	0.87 (0.28-3.76)	1.0	1.0
L5	MRI	0.27 (0.12-0.46)	0.68 (0.49-0.83)	0.8	0.9
S1	MRI	0.17 (0.06-0.35)	0.81 (0.63-0.93)	0.9	1.0

### Deep tendon reflex tests

Deep tendon reflex tests were conducted to establish hypo-reactivity or complete absence. 3 of the reviewed studies evaluated patella reflex or knee jerk while 4 examined the accuracy of the Achilles or ankle reflex. Again, most of the studies did not provide a detailed explanation regarding test execution and definition of positivity. The most recent study (Iversen et al, 2013) reported the highest sensitivity of patella reflex (confidence interval 95%) (0.67 (0.21-0.94)) in detecting L4 nerve root impingement with a relatively good specificity of 0.83 (0.75-0.89) though this was slightly lower compared to a 0.90 (0.89-0.95) specificity rate reported in an earlier study by Suri et al. (2010) [15].

The recent Iversen et al. (2013) study also reported the highest specificity (confidence interval 95%) 0.67 (0.21-0.94) of the Achilles tendon reflex test in detecting lower lumbar (L5S1) nerve root impingement compared to the other 3 studies which investigated the accuracy of the same test. However, the best specificity (confidence interval 95%) 0.93 (0.87-0.97) of the Achilles tendon reflex was found in the much earlier primary study (Vroomen et al, 2002) [10]. A summary of the diagnostic parameters of deep tendon reflex tests is presented in Table 5.

**Table 5:** Diagnostic accuracy of tendon reflex tests

Author (year)	Reference standard	Sensitivity (95% CI)	Specificity (95% CI)	+ LR	- LR
Patella reflex Suri et al. (2010) [15]	MRI	0.32 (0.31-0.53)	0.90 (0.89-0.95)	3.2	1.3
Iversen et al. (2013)	MRI & CT	0.67 (0.21-0.94)	0.83 (0.75-0.89)	4.0	2.5
Coster et al. (2010) [7]	EMG	0.18 (0.10-0.18)	0.66 (0.58-0.71)	0.5	0.8
Achilles reflex Albeck (1996) [13]	Surgery	0.61 (0.47-0.73)	0.63 (0.38-0.84)	1.8	1.6
Vroomen et al. (2002) [10]	MRI	0.14 (0.09-0.21)	0.93 (0.87-0.97)	2.0	1.1
Suri et al. (2011) [21]	MRI	0.33 (0.13-0.59)	0.91 (0.77-0.98)	3.7	1.4
Iversen et al. (2013)	MRI & CT	0.67 (0.21-0.94)	0.60 (0.51-0.69)	1.7	1.8

### Neuro-dynamic tests

The accuracy of neuro-dynamic or provocative tests were also evaluated in most of the reviewed studies, authors in these primary diagnostic accuracy studies (Iversen et al. 2013, Suri et al. 2011 [21], Trainor and Pinnington 2011 [16], Coster et al. 2010 [7], Suri et al. 2010 [15], Bertilson et al. 2010 [14], Lee-Robinson et al. 2010 [2], Majlesi et al. 2008 [20], Rabin 2007 [19], Vroomen et al. 2002 [10], Haldeman et al. 1998 [18], Albeck 1996 [13]) used provocative tests to establish the level of disc herniation and subsequent impingement of the exiting or traversing nerve root and not the response of the lower limb peripheral neural system towards mechanical loading. Similarly, SLR test and Lassegue’s sign were used inter-changeably with one study (Albeck, 1996) [13] describing the later and reporting about the former. The diagnostic performance of the SLR test however had the highest sensitivity of (confidence interval 95%) 0.93 (0.87-0.97) reported in both Albeck, 1996 [13] and Majlesi, 2008 [20] studies. The difference between these two studies being the reference standard where the former used intra-operative findings while the later used MR imaging. On the other hand, a specificity rate of 1.00 (0.48-1.00) for the SLR test was reported in the relatively current Suri et al. (2011) [21] study. The diagnostic parameters of lower limb neuro-dynamic tests are summarized in Table 6.

**Table 6:** Diagnostic accuracy of lower limb neuro-dynamic tests

Type of index test (Author, year)	Reference standard	Sensitivity (95% CI)	Specificity (95% CI)	+LR	-LR
SLR & Lassegu’s sign					
Majlesi (2008) [20]	MRI	0.52 (0.42-0.58)	0.89 (0.79-0.95)	4.7	1.9
Vroomen et al. (2002) [10]	MRI	0.64 (0.56-0.71)	0.57 (0.47-0.66)	1.5	1.6
Albeck (1996) [13]	Surgery	0.84 (0.72-0.92)	0.21 (0.06-0.46)	1.1	1.3
Haldeman (1988) [18]	CT and electro-diagnostics	0.37 (0.19-0.58)	0.78 (0.67-0.87)	1.7	1.2
Suri et al. (2010) [15]	MRIss	0.64 (0.47-0.82)	0.48 (0.45-0.50)	1.2	1.3
Coster et al. (2010) [7]	EMG	0.44 (0.38-0.52)	1.00 (0.48-1.00)	0.4	1.8
Suri et al. (2011) [21]	MRI	0.29 (0.28-0.32)	0.57 (0.48-058)	0.7	0.8
Rabin (2007) [19]	MRI	0.67 (0.53-0.79)	0.43 (0.38-0.46)	1.0	1.3
Slump test					
Majlesi (2008) [20]	MRI	0.84 (0.74-0.90)	0.83 (0.73-0.90)	5.0	5.2
Trainor & Pinnington (2011) [16]	MRI	1.00 (0.40-1.00)	0.83 (0.52-0.98)	5.9	0.8

## Discussion

The current review evaluated 12 primary diagnostic accuracy studies that specifically assessed the performance of various individual clinical neurological tests in detecting nerve root impingement. Different from previous reviews [11–13], we did not consider disc herniation as the cause of nerve root impingement and subsequent radiculopathy. A meta-analysis of pooled data for individual tests was not performed due to heterogenity of the included studies.

The current review analysed the accuracy of index tests for diagnosing lumbo-sacral radiculopathy (sensory, motor, reflex and neuro-dynamic) by comparing them to MR imaging, electro-diagnostics or intra-operative findings either in generally detecting nerve root impingement at mid-lumbar (L2-L4) or lower-lumbar (L4-S1) or at specific segmental nerve root levels (L2, L3, L4, L5, S1).

All the studies after the year 2000, that evaluated the diagnostic performance of sensory testing, used MR imaging as a reference standard. However, the oldest study by Albeck (1996) [13] which compared clinical assessment with surgical findings, demonstrated the best sensitivity (confidence interval 95%) 0.61 (0.47-0.73) with a moderate specificity of 0.63 (0.38-0.84). Higher specificity in this study may be attributed to the fact that patients who are scheduled for surgery are carefully selected compared to those whom surgey is not contemplated. Hence the probability of a positive index test result becomes relatively higher in the surgical than imaging group. The results of the reviewed studies indicate that sensory testing of superficial soft touch and superficial pain are very specific and could therefore be used to rule in the diagnosis of lumbo-sacral radiculopathy among patients presenting with low back and radiating leg symptoms.

Motor tests evaluated in the reviewed studies were mostly functional tests of heel walk, heel raise, sit-to-stand, and resisted isometric contractions for hip flexion, knee extension, great toe extension, ankle dorsi- and planter flexion. The test in all studies was determination of paresis or muscle weakness. Sensitivity was poor to moderate, ranging from 0.13 (0.04-0.31), in the study of Bertilson et al. (2010) [14] to 0.61 (0.36-0.83), in the study of Suri et al. (2011) [21]. The clinical implication of these findings is that motor tests are not ideal for ruling out the diagnosis of lumbo-sacral radiculopathy. The highest specificity was reported in the Suri et al. (2011) [21] for detecting S1 nerve root impingement. A clear description of the actual execution of motor tests, which permits duplication, was provided in the Bertilson (2010) [14] study.

Deep tendon reflex testing focused on evaluation of the patella and Achilles’ tendon reflexes. Generally, in the studies where reflex testing was included, diagnostic performance of reflex tests across the studies was notably good with specificity ranging from 0.60 (0.51-0.69) in the recent Iversen et al. study to 0.93 (0.87-0.97) in the Vroomen (2002) [10] study. However, the sensitivity was moderate with the highest being 0.67 (0.21-0.94) in the Iversen (2013) study. Therefore the results of this review present evidence for use of deep tendon reflex tests as confirmatory tests in the diagnosis of lumbo-sacral radiculopathy. However, index test procedure, together with the cut-off values for positivity, were not provided in some of the studies, and where provided, there were outright procedural variations.

There seem to be a disconnect among researchers regarding the diagnostic utility of lower limb neuro-dynamic tests which include the SLR test for the sacral plexus and the femoral nerve stretch test for the lumbar plexus. In some studies, these tests were intended to detect the presence of disc herniation and subsequent nerve root compression [7], and in some [10, 16] studies they were proposed to test mechanical sensitivity of the femoral and sacral plexii. Also, the procedural difference between the SLR test and Lassegue’s sign is not clear to some authors of primary diagnostic test accuracy studies. There is thus a high probability that such variations would negatively impact on the reported diagnostic performance of the neuro-dynamic tests. A good sensitivity and specificity 1.00 (0.40-1.00) and 0.83 (0.52-0.98) respectively was reported in the Trainor & Pinnington (2011) [16] study with the rest of the studies recording a poor and moderate diagnostic performance. Therefore in light of these findings, lower limb neuro-dynamic tests (FNST and SLRT) are more sensitive than specific hence ideal for ruling out the diagnosis of lumbo-sacral radiculopathy.

In this review, the diagnostic accuracy of most clinical neurological tests range from low to moderate. This finding may stem from several factors ranging from variations in operational case definition of the target condition, outcome of clinical testing, that is, detection of radiculopathy due to disc-related nerve root compression among others.

The outcome of previous systematic reviews on diagnostic accuracy of clinical neurological testing could be questioned due to inconsistencies in specific objectives of diagnostic tests for the primary study selection, and therefore the criteria used to select studies.

Verification bias may also contribute towards the minimal utility of clinical neurological tests reported since the commonly utilized reference standard is MR imaging whose value and accuracy is known only in detecting visible structural nerve root impingement which does not necessarily mediate radicular symptoms yet the evaluated index tests are intended to detect radicular symptoms.

Another contributing factor to the rather poor performance of sensory tests is the variability of dermatomal maps for sensory testing. These tests are guided by published dermatome maps indicating the cutaneous fields of the suspected spinal nerve roots, however, there are reported variations among these maps [19, 21]. Dermatomes are also known to overlap and vary across individuals due to possible extra-dural anomalies where two pairs of nerve roots may arise from a single dural sleeve or extra-dural anastomosis [22].

While clinical neurological tests remain a vital component of the initial diagnostic work-out of patients suspected of radiculopathy, and for researcher and clinicians to establish their actual clinical utility, a common ground must be reached in terms of operational definition of the target condition, the index test outcome and the homogeneity of reviewed studies. This would improve the reported accuracy and ultimately the diagnostic credibility of clinical tests.

## Conclusion

Sensory testing has moderate sensitivity in the detection of lumbo-sacral radiculopathy and prior knowledge of MRI results is a source of bias in sensory testing. This review highlights the inconsistencies in execution of motor tests and grading of test results, such methodological di-similarities could be attributed to the variations in motor tests sensitivities as reported in the primary diagnostic studies analysed in this review. Similarly, SLR test and Lassegue’s sign have been used interchangeably with variation on the expected diagnostic outcome on whether they detect IVD prolapse and subsequent nerve root impingement or hypersensitivity of the lumbar and sacral plexii to mechanical loading. There is however an acceptable level of consistency and similarities in execution and reporting of deep tendon reflex tests which in this review showed good sensitivity in detecting lumbo-sacral radiculopathy. However, in clinical practice, the diagnosis of lumbo-sacral radiculopathy should always be arrived at through consolidation of sensory, motor and deep tendon reflex test results and not isolated single test results.

## Abbreviations

CT scan: Computed Tomograpy Scan
EMG: Electro-myography
FNST: Femoral Nerve Stretch Test
IVD: Inter-vertebral Disc
LBP: Low back pain
LLNDT: Lower Limb Neuro-dynamic Test
MRI: Magnetic Resonance Imaging
PICO: Patient, Indicator, Comparison, Outcome
QAUDAS: Qualify Assessment of Diagnostic Accuracy Studies
RICs: Resisted Isometric Contractions
SLRT: Straight Leg Raise Test
